# Social Network Analysis of COVID-19 Sentiments: 10 Metropolitan Cities in Italy

**DOI:** 10.3390/ijerph19137720

**Published:** 2022-06-23

**Authors:** Gabriela Fernandez, Carol Maione, Harrison Yang, Karenina Zaballa, Norbert Bonnici, Jarai Carter, Brian H. Spitzberg, Chanwoo Jin, Ming-Hsiang Tsou

**Affiliations:** 1 Metabolism of Cities Living Lab, Center for Human Dynamics in the Mobile Age, Department of Geography, San Diego State University, San Diego, CA 92182, USA; carol.maione@polimi.it (C.M.); hyang5959@sdsu.edu (H.Y.); nikazaballa@gmail.com (K.Z.); norbert@bonnici.mt (N.B.); jarai.carter@gmail.com (J.C.); spitz@sdsu.edu (B.H.S.); cjin@sdsu.edu (C.J.); mtsou@sdsu.edu (M.-H.T.); 2 Department of Management, Economics, and Industrial Engineering, Politecnico di Milano, 20156 Milan, Italy; 3 Malta Critical Infrastructure Protection Directorate, 1532 Valletta, Malta; 4 Smart Lab, Procter & Gamble, Champaign, IL 61820, USA; 5 Department of Communication, San Diego State University, San Diego, CA 92182, USA

**Keywords:** COVID-19, Italy, sentiment analysis, Twitter, fear, anger, joy, metropolitan cities, social network analysis

## Abstract

The pandemic spread rapidly across Italy, putting the region’s health system on the brink of collapse, and generating concern regarding the government’s capacity to respond to the needs of patients considering isolation measures. This study developed a sentiment analysis using millions of Twitter data during the first wave of the COVID-19 pandemic in 10 metropolitan cities in Italy’s (1) north: Milan, Venice, Turin, Bologna; (2) central: Florence, Rome; (3) south: Naples, Bari; and (4) islands: Palermo, Cagliari. Questions addressed are as follows: (1) How did tweet-related sentiments change over the course of the COVID-19 pandemic, and (2) How did sentiments change when lagged with policy shifts and/or specific events? Findings show an assortment of differences and connections across Twitter sentiments (fear, anger, and joy) based on policy measures and geographies during the COVID-19 pandemic. Results can be used by policy makers to quantify the satisfactory level of positive/negative acceptance of decision makers and identify important topics related to COVID-19 policy measures, which can be useful for imposing geographically varying lockdowns and protective measures using historical data.

## 1. Introduction

In 2020, lives changed dramatically due to the outbreak of coronavirus disease, known as SARS-CoV-2 or COVID-19. Since then, the scientific community has shown remarkable success in determining the short- and long-term health impacts of the pandemic, especially those associated with a higher risk of fatality and pre-existing conditions [1,2,3]. Lesser known are the effects on mental health and behaviors linked to lockdown measures and containment policies, fear of contagion and ultimately death, chronic anxiety, and distress, that superimposed unprecedented restrictions on individual freedom. Studies have associated these conditions with an increase in reported psychological disorders and depression, especially across younger groups [4]. Furthermore, when combined with pre-existing conditions, psychological disorders could lead to severe psychiatric consequences and suicidal behavior [5].

Previous studies demonstrate that social media are a gold mine for investigating the societal response to the pandemic outbreak [6,7,8]. During times of emergency, in fact, social media platforms become primary communication channels that ordinary people use to form and share opinions and beliefs around a certain topic and monitor real-time emotions [9,10]. People rely more and more on social media sites such as Twitter or Facebook to connect to the outer world, showcasing emotions and sentiments linked to COVID-19 [11].

This study deals with an extensive analysis of emotions contained in Twitter posts during the first wave of the pandemic. For this study, 4,227,882 million coronavirus-related tweets between 2 March 2020 and 15 June 2020 were collected for 10 Italian cities during the period of March 2020 to June 2020. The research aimed to explore regional variations in the social response to COVID-19 across Italy through the analysis of three sentiment emotions: fear, anger, and joy. The aim of this study was to analyze the national spread of COVID-19 across Italy (north, center, south, and islands). The striking spatial unevenness of COVID-19 suggests that the infection has hit economic core locations harder, and this raises questions about whether, and how, the geography of the disease is connected to the economic base of localities and policy. This study viewed 10 metropolitan cities (i.e., widely affected regions) while analyzing sentiments and perceptions across the north, center, south, and island regions of Italy to identify geospatial patterns during the COVID-19 pandemic, which follow the lines of the local economic landscape, culture, resources, funding, infrastructure, policy, and spatial economic forces. The COVID-19 restrictions and containment measures aimed at preventing the mobility and interaction of people inside and across these areas and included, for instance, the suspension of social gatherings, cultural and religious activities, and limitations on restaurants, just to name a few, and had an impact on the people’s well-being, perceptions, and sentiments across the country. This study aimed at presenting a data-driven approach to exploring the spatial-temporal patterns of the pandemic across Italy.

### Related Work

Digital communication media are evolving much faster than our theoretical frameworks for conceptualizing their processes and effects, although the need for theory in social media and big data remains important. From user-generated content on social media, studies have analyzed the public’s thoughts and sentiments on health status, concerns, panic, and awareness related to COVID-19, which can ultimately assist in developing health intervention strategies and designing effective campaigns based on public perceptions [12]. Scholars have taken similar approaches to develop sentiment analysis using Twitter data around the world. One example study of a sentiment analysis and topic modeling research focused on the public perception of the COVID-19 pandemic on Twitter. The study aimed to increase understanding of public awareness of the pandemic trends and uncover meaningful themes of concern posted by Twitter users. The analyses included frequency of keywords, sentiment analysis, and topic modeling to identify and explore discussion topics over time [13]. This work only shows the negative sentiment from some particular topics without analyzing any model in time intervals and is devoid of any sentiment model and analysis. Additionally, the appearance of opinions showed that the approach is stable and viable for understanding public opinion. Other scholars have examined sentiments evoked in tweets only with the judgment of some emerging keywords about COVID-19, examining the top trending topics over time [14]. Furthermore, scholars have presented an issue surrounding public sentiment, leading to the testimony of growth in fear sentiment and negative sentiment [15]. 

Overall, this study focused on Twitter sentiment analysis (fear, anger, and joy) based on geography across Italy during the COVID-19 pandemic and the impact of policy measures/cases, and deaths during the pandemic. The details of the proposed research workflow model are discussed in the next section.

## 2. Materials and Methods

### 2.1. Study Area

Italy’s first confirmed case of COVID-19 was reported on 20 February 2020 in the Province of Lodi, Lombardy. The number of COVID-19 cases escalated quickly, with hundreds of positive cases registered within a few days (with an average mortality rate of 13.7% [16,17]. In the month of March 2020, northern regions accounted for 120% and 568% increases in mortality compared to the same period in 2019 [18].

The pandemic spread rapidly across Italy, and, by 8 March 2020, all regions had reported at least one COVID-19 case or death [19]. From the beginning of the pandemic, cities in the north and central Italy registered more than half of the total COVID-19 cases, with Lombardy (87,417 positive cases), Piedmont (30,314), Veneto (19,105), Tuscany (10,070, and Emilia-Romagna (27,611) recording the highest numbers of positive cases. In particular, the Lombardy region faced the most severe impacts on human lives, putting the region’s health system on the brink of a massive collapse [17].

For this study, major Italian metropolitan cities were explored to understand the interconnections between geographical location, number of COVID-19 cases, and social responses to the pandemic and locally enforced measures. A total of 10 cities were selected across multiple areas: (1) north: Milan, Venice, Turin, Bologna; (2) central: Florence, Rome; (3) south: Naples, Bari; and (4) islands: Palermo, Cagliari.

### 2.2. Data Collection

We collected a total of 4,227,882 COVID-related tweets between 2 March 2020 and 15 June 2020 with the use of the Twitter search application programming interface (api). For the search, we employed a set of Italian and English terms associated with COVID-19. Keywords included: COVID-19; Italy; Sentiment analysis; Twitter; Fear; Anger; Joy; Metropolitan cities; and Social network analysis. Twitter keywords consisted of a set of predefined coronavirus search key terms in both the English and Italian languages. Predefined Twitter search key terms/hashtags in English included: COVID-19, Coronavirus, CoronavirusOutbreak, coronavirusitaly, racism, COVID2019, COVID19italy, Flu, ItalyCoronavirus, Lombardy, Italyquarantine, quarantineItaly, and COVID. Predefined Twitter search key terms in Italian included: razzismo, Italiani all’estero, Influenza, Amuchina, Codogno, Contagiati, Contagio, Coronaviriusitalia, COVID19italia, COVID2019italia, Coronavirusitalia, CoronavirusItalla, Lombardia, zonarossa, focolai, and quarentena.

For data processing, we employed several Python libraries, including Tweepy, Pandas, and BeautifulSoup, that served as bases to create our own script. In particular, the Tweepy library was employed to provide API access to Twitter, while Panda served to handle the data frames, and BeautifulSoup to extract data from HTML and XML files. In addition, we used other libraries, such as re(regular expressions), json, sys, and datatime. Next, we extracted and sorted text and metadata from our Twitter search, paying special attention to possible misspellings.

### 2.3. Data Analysis

Twitter was our main platform for mining public sentiments. In fact, it provides access to a consistent number of public-generated posts and reactions regarding COVID-19. More specifically, our analysis considered sentiments related to fear, anger, and joy. The sentiment analysis was conducted using the NRC Emoticon Lexicon [20,21], a platform scoring 14,182 positive and negative unigrams. Based on this scoring system, we obtained a normalized score for each collected emotion. Finally, the mean sentiment score for fear, anger, and joy was computed across all tweets collected for a given date. The findings were then crossed analyzed with the total number of COVID-19-positive cases, national COVID-19 measures, and user characteristics. 

**Figure 1:**
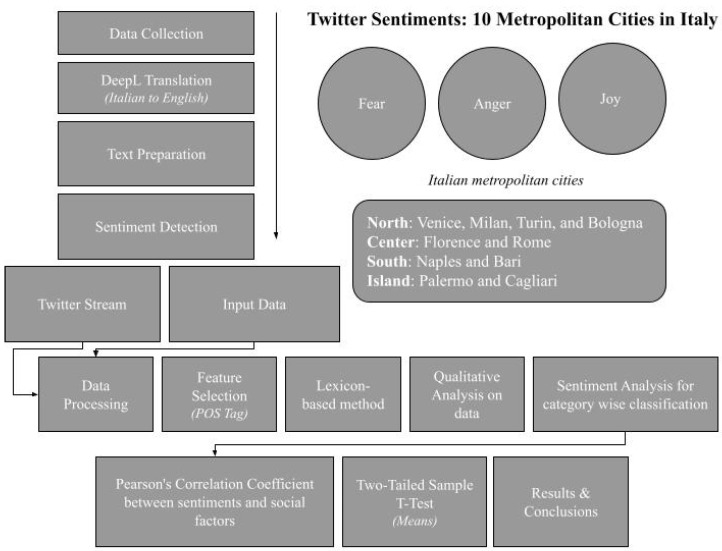
Research work flow model.

Figure 1 shows the research workflow metrics used to develop a sentiment analysis (SA). Over 4 million Twitter tweets related to fear, anger, and joy were analyzed for major Italian metropolitan cities during the first wave of the COVID-19 pandemic. The 10 cities included: (i) north: Milan, Venice, Turin, Bologna; (ii) central: Florence, Rome; (iii) south: Naples, Bari; and (iv) islands: Palermo, Cagliari.

To this end, we selected a number of socioeconomic indicators, such as (1) demographics, such as percentage of people aged 65+, total number of COVID-19 deaths, and cumulative number of COVID-19-positive cases, including percentage of people aged 65 or older, total number of COVID-related deaths, and cumulative number of COVID-19 cases; (2) environmental indicators, such as population density, industry manufacture expenditure, and industry services expenditure; and (3) economic indicators, including GDP per capita, unemployment rate, and intensive care unit (ICU) beds to explore the differences and capacities between the four Italian regions of Italy (north, central, south, and islands. A one-way ANOVA or *t*-test was performed to determine statistically significant differences between the mean values of different Italian regions and assess the influence of each selected indicator.

## 3. Results

Through analyses of sentiments for the COVID-19 Twitterverse at the global, national, regional, and city levels, analyses may reveal important intersections of public events, policies, and public reactions. Such focused analyses become important for the adaptation of health campaigns and policies to the specifics of a given locality. This study explored the impact of fear, anger, and joy during the first wave of COVID-19 in 10 Italian metropolitan cities. This analysis served to explore possible interconnections between such emotions and the enforcement of nation-wide measures to contain the pandemic. 

Scholars have found that mental health emerged during the COVID-19 outbreak, including stress, anxiety, depression, frustration, and uncertainty [22]. In relation to mass quarantines imposed in order to attenuate the spread of COVID-19, common psychological reactions of fear and pervasive community anxiety occurred, which are typically associated with disease outbreaks. These reactions increased with the escalation of new cases together with anxiety-provoking (mis)information provided by media. 

### 3.1. Sentiment Analysis 

Mean sentiment analysis at the national level is presented below. Exact information for the 10 selected cities is presented in Appendix A. Overall, for fear and anger, our results show that the initial response (March–April) was volatile and trending at higher values, with its highest level registered on 25 April 2020. After this date, values decreased, reaching a new low on 4 May 2020, which corresponded with the beginning of Phase II of the pandemic. Later, we registered an increased value for both the fear and anger sentiments. Contrarily, the sentiment values for joy were higher at the early stages of the pandemic and dropped as time passed. Overall, the response for joy was volatile, with a mean value of approximately 1.3%.

#### 3.1.1. Fear

**Figure 2:**
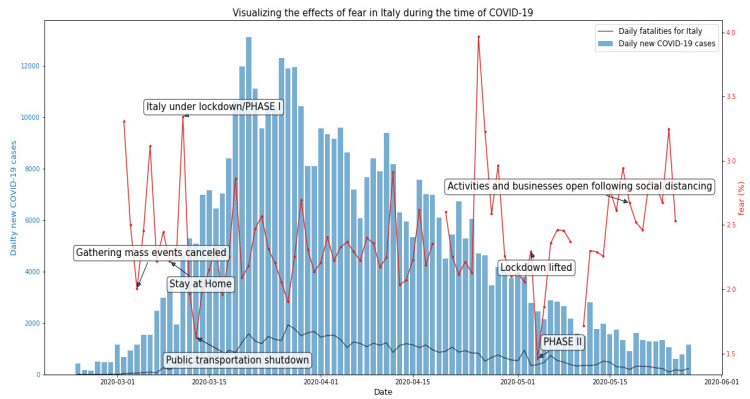
Fear-related tweets in Italy during the first wave of the COVID-19 pandemic.

Figure 2 shows the mean emotion for fear varying over time. Out of the total collected tweets, the highest mean was found to be in the month of April at 2.39% when compared to May at 2.35%, and March at 2.30%, respectively. In this corpus of Italian tweets about COVID-19, 2.34% were related to fear.

#### 3.1.2. Anger

**Figure 3:**
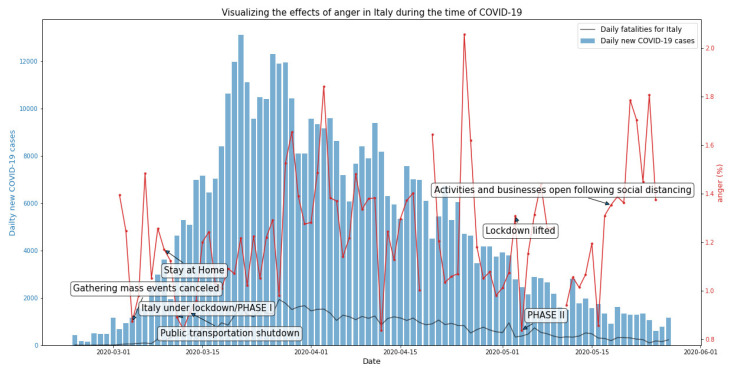
Anger-related tweets in Italy during the first wave of the COVID-19 pandemic.

Figure 3 shows the mean emotion for anger varying over time. Out of the total collected tweets, our analysis shows that the highest frequency of fear sentiments (1.29%) was registered during the month of April 2020, with an average value of 1.19%. A slightly lower occurrence was registered in the months of March (1.13%) and May (1.23%).

#### 3.1.3. Joy

**Figure 4:**
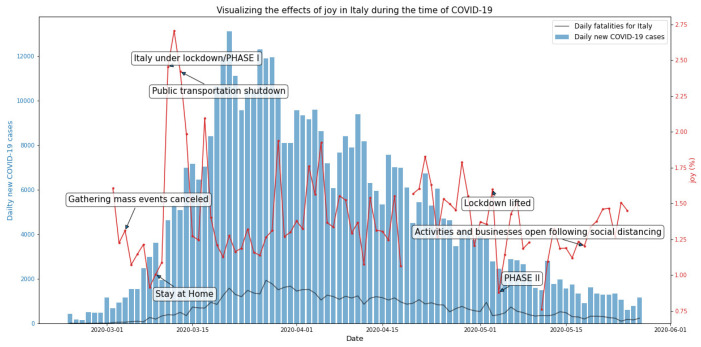
Joy-related tweets in Italy during the first wave of the COVID-19 pandemic.

The highest incidence of joy tweets was registered in the month of March 2020, with a frequency of 1.48% of the total collected tweets, compared to April (1.45%) and May (1.26%). Figure 4 shows a total occurrence of 1.44% tweet frequency associated with joy. 

### 3.2. Cross-City Variations

A two-tailed, two-sampled *t*-test was conducted for each city and emotion to assess possible differences between the mean levels of sentiments in each city under study. From our results, it appeared that sentiments were similarly distributed across cities. With an alpha level value of 0.5R. A cross-city comparison for the three sentiments, fear, anger, and joy, is presented below.

#### 3.2.1. Fear

Table 1 shows the *p*-values of the cross-city comparison for tweets related to fear. When comparing the results between the mean levels of fear, all but three city pairs’ *p*-values were statistically significant (Table 2). The city pair values that proved to be insignificant because their values were within the range of −1.96 and 1.96, were Bologna–Cagliari (−1.802), Florence–Rome (−1.898), and Naples–Palermo (−1.703). Hence, this provides evidence that geographical location influenced most fear sentiment levels. 

#### 3.2.2. Anger 

Table 3 shows the *p*-values of the cross-city comparison for anger tweets. The results for the cross-city comparison for anger show that all but five city pairs recorded statistically significant values (>0.5) (Table 4). 

The difference between the sentiment mean variable for anger in the cities of Bari and Bologna was 10.29 times smaller than expected, while the anger t-statistic values demonstrated a difference in the means which was approximately larger or smaller than expected based on chance. Insignificant values due to the t-statistic, meaning between −1.96 and 1.96, were found between Bari–Bologna (1.695), Bari–Turin (−1.841), Milan–Naples (−0.532), Milan–Venice (1.170), and Naples–Venice (1.326). This provides evidence that geographical location influenced most anger sentiment levels.

#### 3.2.3. Joy

Table 5 shows the *p*-values of the cross-city comparison for joy tweets. When comparing the results between the mean levels of joy, all but two city pairs’ *p*-values were statistically significant (Table 6). The city pair values that proved to be insignificant because their values were within the range of −1.96 and 1.96 were Bari–Rome (0.368) and Florence–Naples (−0.478). This provides evidence that geographical location influenced most joy sentiment levels.

## 4. Discussion

Previous studies conducting a sentiment analysis of the tweets collected during epidemics have shown that fear generally increases during the pre-pandemic period [23,24], becoming a dominant sentiment as users seek who/what to blame for the threats posed to their lives, and eventually decreases over the course of the epidemic, as cases and deaths decrease and more containment measures are put in place [25,26]. In contrast, anger increases as people begin to organize appraisals of who and what to blame for the threats posed to their lives. The tweets in this corpus did not reveal this pattern, at least not within the time span covered in this study. Apart from Sicily COVID-19 tweets, our data showed different patterns for the first wave of the COVID-19 pandemic in Italy. Tweets collected in north and central regions demonstrated rising trends for fear throughout the entire first wave of the pandemic, even when COVID-19 cases started to drop. This trend is possibly associated with uncertainties surrounding the post-pandemic social and economic recovery, including a “new normal” and the prospect of further COVID-19 waves. While fear is a primary motivator, studies showed that it only plays functional roles in health when coupled with trust in the existing healthcare infrastructure. In our study, fear presented the highest values during the last week of April in Italy’s central regions, when several businesses were retrieving their operations. Like previous studies [27], we found that fear was central to social media communication. COVID-19 tweets, in fact, have rapidly spread within topic networks, with fear and stress being main topic themes, especially in association with China [28]. According to another study, over half of COVID-19-related tweets posted in the month of January, 2020, contained fear emotions. This frequency dropped in early April to under 30%, while anger went from 13–14% in January, 2020, to over 30% in early April [23]. 

Notably, our results show an increase in anger tweets from the last week of May in the islands, following the lift of lockdown measures and consequent re-opening of traveling. A study on anger found that this sentiment is far more important in mobilizing public and political action, as well as triggering the propagation of negative news among society, compared to the other sentiments [29]. Fear plays similar influential roles in social media, being uniquely capable of creating cascading and contagious communications [30]. For example, another study on sentiment analysis conducted in China showed similar shifts in the emotions over the course of the pandemic [31]: “as the COVID-19 epidemic began to spread throughout the country after 20 January 2020, the public eased their concerns and fears caused by their uncertainty toward and ignorance of the epidemic and responded to the epidemic with a more objective attitude” [24].

Findings demonstrated a connection between: Exposure to news or events and how this produces effects on the feelings of the population. Scholars have shown that when everyday news was perceived as more negative, subjects experienced more negative effects and fewer positive effects. Additionally, research indicates that people report more negative effects when negative news items are personally relevant [32]. Other studies have underlined the role of emotion in susceptibility to believing fake news. There is a connection between emotion and fake news in which self-reported use of emotion is positively associated with belief in fake news, inducing reliance on emotion and a greater belief in fake news stories compared to a control group or to inducing reliance on reason [33]. 

Psychological processes (emotional, perceptive, and cognitive) produce effects on the manifestation of feelings (positive and negative) when exposed to news events. Studies have shown that emotion has a substantial influence on the cognitive process in humans, including perception, attention, learning, memory, reasoning, and problem solving. As a result, emotion has a strong connection to cognitive influences, perception, and memory, which possibly played a role during the pandemic [34]. 

The study explored 10 metropolitan case study cities based on geography in the north, center, south, and island regions of Italy. We selected 10 Italian cities as a representative sample of a population, seeking to accurately reflect the characteristics of the larger group. Hence, the 10 representative selected cities that were strongly hit during the first wave of the pandemic are considered to have strong political and geographic territorial influences that were reflected in their online expressions of emotion [17,19]. Societal and cultural factors associated with the Italian context, such as traditions, beliefs, perceptions, attitudes, and behaviors, may correlated with the type of sentiment and the way emotion is expressed by the population based on time. Although sentiment analysis is considered an effective method for identifying people’s opinions in mass, the classification errors of standard systems affect the results of the sentiment analysis and may reflect inaccurate analysis. Also, sentiment analysis provides some sense of people’s opinions, and the biases reflected in tweets may in turn mislead users and policy-makers and cause them to make erroneous decisions. Furthermore, societal cultural factors in Italy may have had an impact on their emotions based on socio-economic factors such as age, education, literacy, political preference, viewpoint, family member’s health, own health, or economic/financial burden during the COVID-19 pandemic [35,36].

## 5. Conclusions

To the best of our knowledge, this is the first study analyzing spatial differences in the social response to COVID-19 in Italy using Twitter data. The study offers insights into the distribution of emotions (fear, anger, and joy) related to the spread of COVID-19 across cities located in the north, center, south, and islands of Italy. The study employed a sentiment analysis to further the understanding of emotions contained in user tweets in response to specific measures and milestones of the lockdown and subsequent phases during the months of March to June 2020. Findings demonstrate a connection between exposure to news and/or significant policy measure events, and how this produces effects on the feelings of the population. In addition, the emotional, perceptive, and cognitive psychological processes produce effects on the manifestation of feelings (positive and negative) when exposed to news events. 

Scholars have found that there was a rapid worldwide spread of deleterious socioeconomic and psychological impact of the COVID-19 pandemic. A number of psychological problems and important consequences in terms of mental health, such as stress, anxiety, depression, frustration, and uncertainty, during COVID-19 infection, have been additionally documented together with the most relevant psychological reactions of the general population related to the COVID-19 pandemic. The results of this study can allow decision makers to take a step back and re-design institutional communication strategies related to changes in health policies that are aimed at generating positive feelings in the population. Understanding the effects that information produces on the perception and feelings of the population regarding certain events that affect them provides a potential information resource for adjusting health campaigns. Finally, strategies can be proposed that mitigate the appearance of negative feelings in the population as a result of being exposed to certain events/news associated with new political initiatives. 

Limitations of this study pertain to the selection of the sample size, including the platform used, and the data collection timeline. Moreover, t-statistics represent a relatively static representation of regional differences. Multivariate time-series statistics might provide better a more dynamic approach to understanding such processes. 

Future studies should analyze the specific numerical levels of the sentiment for emotions, alongside any theorized or developed baselines for certain levels of sentiment. Such an analysis could find commonalities in emotional word usage related to statistically significant differences in sentiment levels. Moreover, future research should look more at why some people are more affected by negative news than other news. Finally, in order to develop better predictive models in the future, we suggest including variables that further reflect lockdown levels of individual regions over time. Such variables could be made from close examination of adopted policies, as well as be derived from citizen–government perception–behavior surveys, environmental impacts, finance, and/or management. 

## Figures and Tables

**Table 1:** Fear *p*-values.

Fear T-Stats	Bari	Bologna	Cagliari	Florence	Milan	Naples	Palermo	Rome	Turin
Bologna	9.56 × 10^−3^								
Cagliari	2.21 × 10^−4^	7.15 × 10^−2^ *							
Florence	7.68 × 10^−54^	2.91 × 10^−126^	8.27 × 10^−79^						
Milan	7.93 × 10^−7^	1.55 × 10^−30^	4.28 × 10^−21^	1.64 × 10^−121^					
Naples	3.52 × 10^−19^	1.36 × 10^−58^	3.23 × 10^−38^	5.30 × 10^−35^	2.02 × 10^−29^				
Palermo	1.80 × 10^−10^	4.54 × 10^−27^	8.37 × 10^−24^	3.24 × 10^−23^	1.00 × 10^−4^	8.86 × 10^−2^ *			
Rome	8.10 × 10^−59^	5.88 × 10^−168^	1.05 × 10^−85^	5.76 × 10^−2^ *	0	2.00 × 10^−65^	2.23 × 10^−25^		
Turin	2.44 × 10^−4^	4.72 × 10^−19^	7.16 × 10^−16^	5.22 × 10^−98^	1.18 × 10^−2^	2.96 × 10^−25^	1.96 × 10^−6^	2.46 × 10^−200^	
Venice	4.22 × 10^−28^	1.78 × 10^−73^	6.98 × 10^−49^	6.99 × 10^−13^	4.84 × 10^−45^	1.38 × 10^−5^	6.34 × 10^−6^	1.14 × 10^−15^	5.31 × 10^−40^

Note: characteristics: total COVID-19 cases: total number of COVID-19 cases; daily COVID-19 cases: total number of COVID-19 cases per day; total COVID-19 deaths: total number of deaths; daily COVID-19 deaths: total number of deaths per day; anger: total number of Twitter tweets related to anger; joy: total number of Twitter tweets related to joy; fear: total number of Twitter tweets related to fear. * means that *p*-value is less than 0.05 but more than or equal to 0.01.

**Table 2:** Fear t-static values.

Fear T-Stats	Bari	Bologna	Cagliari	Florence	Milan	Naples	Palermo	Rome	Turin
Bologna	−2.591	-	-	-	-	-	-	-	-
Cagliari	−3.694	−1.802 *	-	-	-	-	-	-	-
Florence	15.463	23.926	18.831	-	-	-	-	-	-
Milan	4.938	11.491	9.432	−23.463	-	-	-	-	-
Naples	8.954	16.148	12.939	−12.345	11.263	-	-	-	-
Palermo	6.378	10.777	10.063	−9.927	3.891	−1.703 *	-	-	-
Rome	16.195	27.691	19.676	−1.898 *	57.531	17.086	10.415	-	-
Turin	3.668	8.920	8.071	−21.020	−2.519	−10.384	4.758	−30.224	-
Venice	10.996	18.142	14.712	−7.180	14.088	4.347	4.515	−8.012	13.240

Note: characteristics: total COVID-19 cases: total number of COVID-19 cases; daily COVID-19 cases: total number of COVID-19 cases per day; total COVID-19 deaths: total number of deaths; daily COVID-19 deaths: total number of deaths per day; anger: total number of Twitter tweets related to anger; joy: total number of Twitter tweets related to joy; fear: total number of Twitter tweets related to fear. * means that *p*-value is less than 0.05 but more than or equal to 0.01.

**Table 3:** Anger *p*-values.

Fear T-Stats	Bari	Bologna	Cagliari	Florence	Milan	Naples	Palermo	Rome	Turin
Bologna	7.830 × 10^−25^	-	-	-	-	-	-	-	-
Cagliari	5.292 × 10^−29^	6.132 × 10^−4^	-	-	-	-	-	-	-
Florence	6.632 × 10^−11^	2.595 × 10^−110^	4.667 × 10^−79^	-	-	-	-	-	-
Milan	4.309 × 10^−2^	2.601 × 10^−87^	1.024 × 10^−57^	6.280 × 10^−22^	-	-	-	-	-
Naples	9.007 × 10^−2^ *	4.146 × 10^−69^	4.051 × 10^−52^	3.790 × 10^−17^	5.945 × 10^−1^ *	-	-	-	-
Palermo	2.037 × 10^−14^	8.574 × 10^−96^	7.888 × 10^−79^	1.196 × 10^−2^	2.947 × 10^−21^	4.615 × 10^−19^	-	-	-
Rome	1.307 × 10^−9^	1.267 × 10^−142^	1.333 × 10^−83^	2.025 × 10^−2^	4.162 × 10^−84^	5.727 × 10^−25^	5.877 × 10^−6^	-	-
Turin	6.569 × 10^−2^ *	7.627 × 10^−38^	4.643 × 10^−34^	1.977 × 10^−44^	4.526 × 10^−20^	2.052 × 10^−11^	4.790 × 10^−39^	5.676 × 10^−79^	-
Venice	1.717 × 10^−2^	1.302 × 10^−64^	1.893 × 10^−52^	8.071 × 10^−10^	2.419 × 10^−1^ *	1.849 × 10^−1^ *	2.760 × 10^−13^	1.336 × 10^−9^	5.429 × 10^−12^

Note: characteristics: total COVID-19 cases: total number of COVID-19 cases; daily COVID-19 cases: total number of COVID-19 cases per day; total COVID-19 deaths: total number of deaths; daily COVID-19 deaths: total number of deaths per day; anger: total number of Twitter tweets related to anger; joy: total number of Twitter tweets related to joy; fear: total number of Twitter tweets related to fear. * means that *p*-value is less than 0.05 but more than or equal to 0.01.

**Table 4:** Anger *t*-statistic values.

Fear T-Stats	Bari	Bologna	Cagliari	Florence	Milan	Naples	Palermo	Rome	Turin
Bologna	−10.293	-	-	-	-	-	-	-	-
Cagliari	−11.182	−3.426	-	-	-	-	-	-	-
Florence	6.530	22.335	18.862	-	-	-	-	-	-
Milan	2.023	19.831	16.044	−9.626	-	-	-	-	-
Naples	1.695 *	17.582	15.213	−8.420	−0.532 *	-	-	-	-
Palermo	7.650	20.785	18.825	2.513	9.468	8.923	-	-	-
Rome	6.068	25.480	19.426	−2.322	19.432	10.321	−4.531	-	-
Turin	−1.841 *	12.864	12.178	−13.986	−9.175	−6.702	−13.077	−18.821	-
Venice	2.383	16.981	15.260	−6.144	1.170 *	1.326 *	−7.306	−6.063	6.894

Note: characteristics: total COVID-19 cases: total number of COVID-19 cases; daily COVID-19 cases: total number of COVID-19 cases per day; total COVID-19 deaths: total number of deaths; daily COVID-19 deaths: total number of deaths per day; anger: total number of Twitter tweets related to anger; joy: total number of Twitter tweets related to joy; fear: total number of Twitter tweets related to fear. * means that *p*-value is less than 0.05 but more than or equal to 0.01.

**Table 5:** Joy *p*-values.

Fear T-Stats	Bari	Bologna	Cagliari	Florence	Milan	Naples	Palermo	Rome	Turin
Bologna	6.10 × 10^−94^	-	-	-	-	-	-	-	-
Cagliari	3.29 × 10^−27^	1.26 × 10^−9^	-	-	-	-	-	-	-
Florence	4.69 × 10^−3^	4.16 × 10^−116^	3.80 × 10^−24^	-	-	-	-	-	-
Milan	3.29 × 10^−45^	6.96 × 10^−48^	9.81 × 10^−3^	1.92 × 10^−73^	-	-	-	-	-
Naples	8.87 × 10^−4^	7.99 × 10^−131^	7.99 × 10^−25^	0.632 *	5.05 × 10^−116^	-	-	-	-
Palermo	9.80 × 10^−6^	2.34 × 10^−70^	1.26 × 10^−14^	1.44 × 10^−2^	2.95 × 10^−23^	2.25 × 10^−2^	-	-	-
Rome	0.713 *	5.71 × 10^−215^	2.27 × 10^−43^	1.29 × 10^−9^	0	1.78 × 10^−18^	6.21 × 10^−13^	-	-
Turin	0.044	6.88 × 10^−148^	7.48 × 10^−30^	0.133	4.73 × 10^−153^	0.018	1.40 × 10^−4^	1.08 × 10^−8^	-
Venice	4.42 × 10^−21^	8.76 × 10^−51^	5.19 × 10^−6^	5.51 × 10^−20^	1.13 × 10^−6^	8.48 × 10^−23^	3.54 × 10^−7^	1.50 × 10^−70^	5.92 × 10^−32^

Note: characteristics: total COVID-19 cases: total number of COVID-19 cases; daily COVID-19 cases: total number of COVID-19 cases per day; total COVID-19 deaths: total number of deaths; daily COVID-19 deaths: total number of deaths per day; anger: total number of Twitter tweets related to anger; joy: total number of Twitter tweets related to joy; fear: total number of Twitter tweets related to fear. * means that *p*-value is less than 0.05 but more than or equal to 0.01.

**Table 6:** Joy t-statistics values.

Fear T-Stats	Bari	Bologna	Cagliari	Florence	Milan	Naples	Palermo	Rome	Turin
Bologna	−20.584	-	-	-	-	-	-	-	-
Cagliari	−10.809	6.074	-	-	-	-	-	-	-
Florence	−2.828	22.927	10.142	-	-	-	-	-	-
Milan	−14.126	14.548	2.583	−18.138	-	-	-	-	-
Naples	−3.324	24.369	10.295	−0.478 *	22.905	-	-	-	-
Palermo	−4.422	17.743	7.712	−2.448	9.938	−2.282	-	-	-
Rome	0.368 *	31.393	13.828	6.069	63.779	8.771	7.197	-	-
Turin	−2.015	25.938	11.358	1.504	26.367	2.365	3.808	−5.718	-
Venice	−9.425	14.994	4.558	−9.154	4.868	−9.830	−5.092	−17.768	−11.767

Note: characteristics: total COVID-19 cases: total number of COVID-19 cases; daily COVID-19 cases: total number of COVID-19 cases per day; total COVID-19 deaths: total number of deaths; daily COVID-19 deaths: total number of deaths per day; anger: total number of Twitter tweets related to anger; joy: total number of Twitter tweets related to joy; fear: total number of Twitter tweets related to fear. * means that *p*-value is less than 0.05 but more than or equal to 0.01.

**Figure A1:**
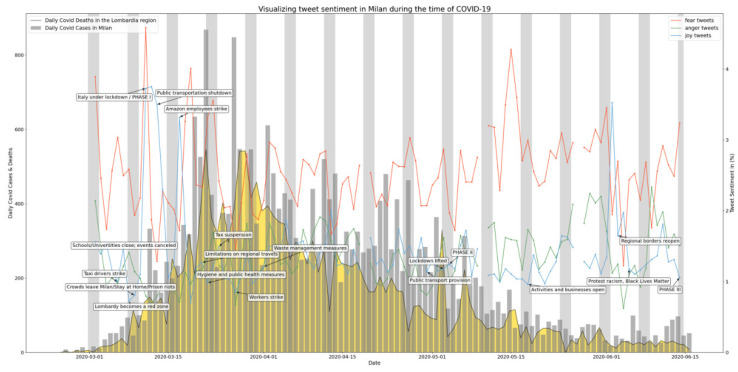
Visualizing Twitter sentiment in Milan during the time of COVID-19.

**Figure A2:**
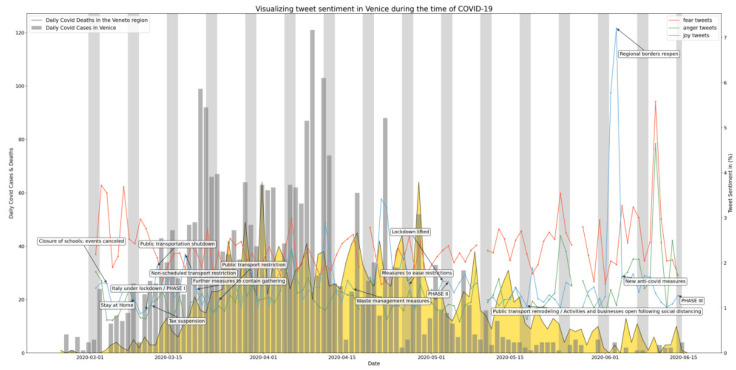
Visualizing Twitter sentiment in Venice during the time of COVID-19.

**Figure A3:**
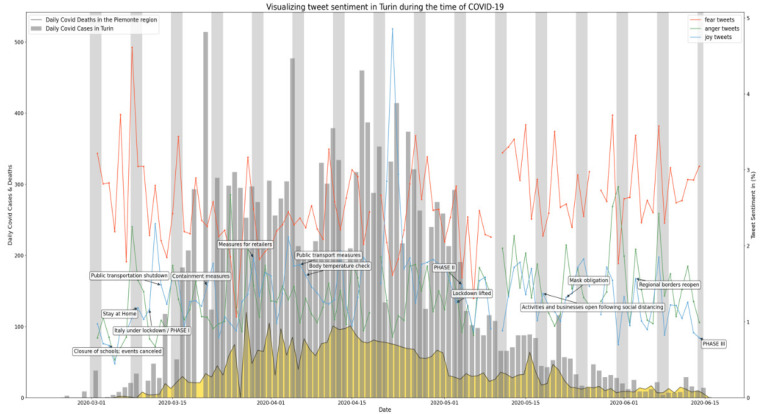
Visualizing Twitter sentiment in Turin during the time of COVID-19.

**Figure A4:**
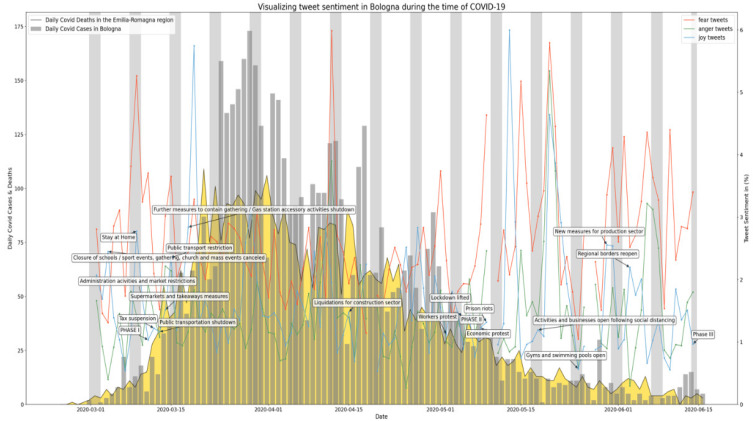
Visualizing Twitter sentiment in Bologna during the time of COVID-19.

**Figure A5:**
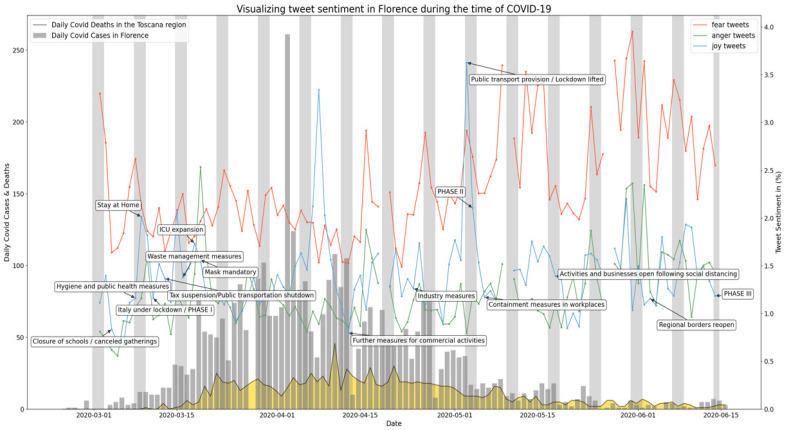
Visualizing Twitter sentiment in Florence during the time of COVID-19.

**Figure A6:**
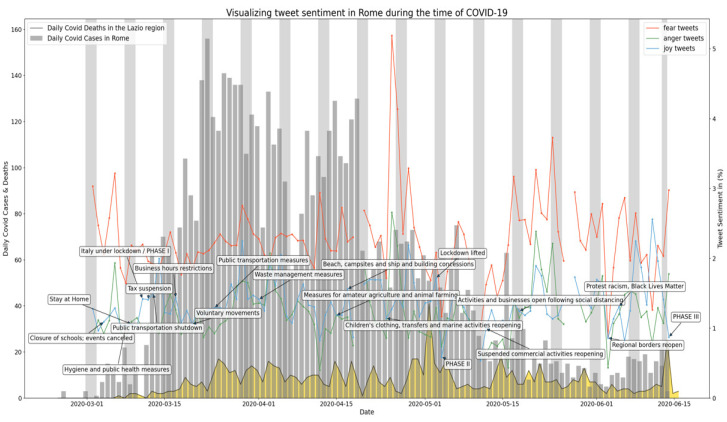
Visualizing Twitter sentiment in Rome during the time of COVID-19.

**Figure A7:**
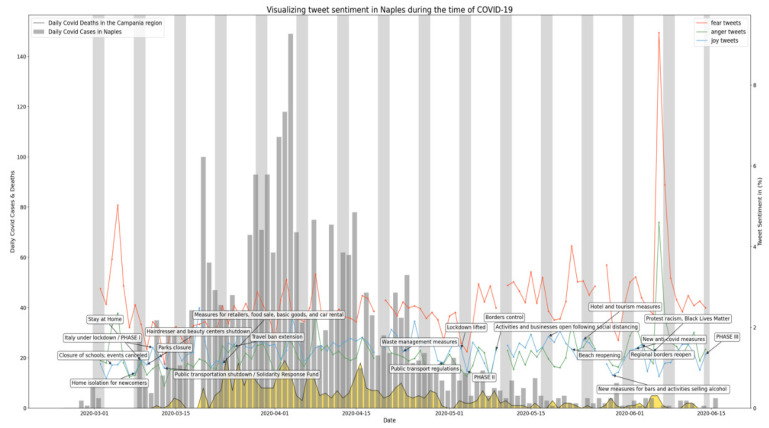
Visualizing Twitter sentiment in Naples during the time of COVID-19.

**Figure A8:**
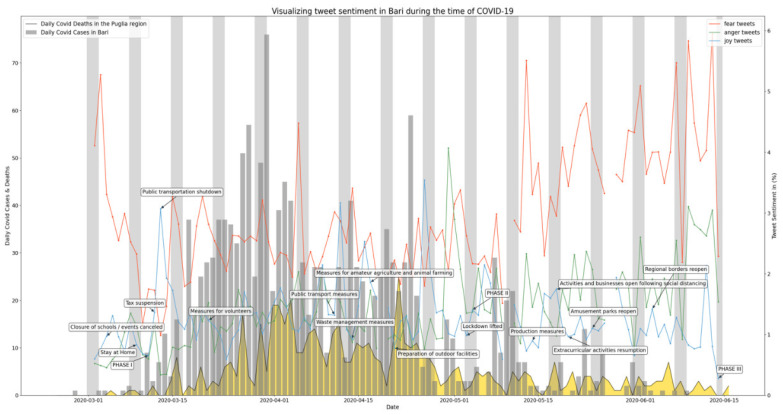
Visualizing Twitter sentiment in Bari during the time of COVID-19.

**Figure A9:**
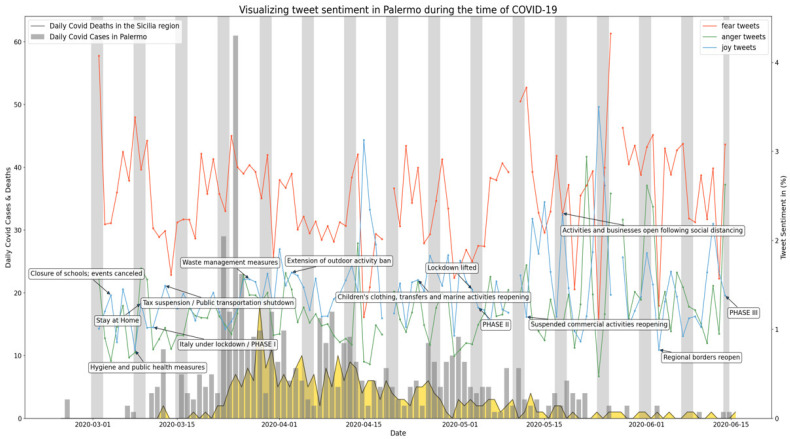
Visualizing Twitter sentiment in Palermo during the time of COVID-19.

**Figure A10:**
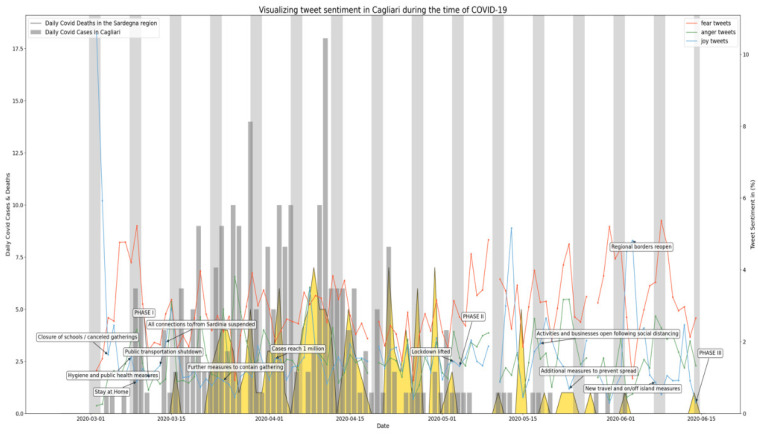
Visualizing Twitter sentiment in Cagliari during the time of COVID-19.

## Data Availability

Data supporting reported results can be found, including links to publicly archived datasets analyzed or generated during the study. All code used in this study to generate the Twitter translation and sentiment analysis is freely available in our GitHub repository: https://github.com/HDMA-SDSU/Translate-Tweets (accessed on 16 June 2022). Please visit our Social Response to COVID-19 in Italy Story Maps here: https://storymaps.arcgis.com/stories/74c499d5ac0a46ffbbc2b28acfa05102 (accessed on 16 June 2022).

## Appendix A

### Appendix A.1. Northern Regions

#### Appendix A.1.1. Milan, Lombardy

##### Region Outlook

The Lombardy region is home to a sixth of the Italian population and located in the north of Italy. The region comprises 20 provinces including the metropolitan city of Milan, which more recently became the epicenter of the country’s outbreak. On 23 February, urgent measures regarding the containment and management of COVID-19 took place, and the Lombardy region was identified as a code-red zone (11 municipalities with confirmed positive cases). On 29 February, specific measures were given to people who recently traveled to China. On 10 March, all travels in and out of a municipality were restricted, and train stations, airports, and cruise ships remained closed. Phase I, initiated on 11 March, banned all public gatherings, universities, schools, church and mass events, museums, and exhibitions, including weddings and funerals, and sporting events. Several labor strikes took place, including strikes by taxi drivers (6 March), Amazon employees (17 March), postal service workers (27 May), and workers regarding COVID-19 safety issues (27 March), as well as prison riots regarding pandemic-related containment measures (9 March). A “Stay at Home” campaign went into effect in the north of Italy to take measures to stop the spread of the virus and to prevent hospitals from being overloaded, including suspension of public transportation (13 March). On 6 April, further measures for the management of the epidemiological emergency took effect, including outdoor markets. Provisions for local public transport took place on 30 April, while the lockdown ended on 3 May. In Phase II, initiated on 4 May, people were allowed to visit loved ones, and specific areas were opened with public safety measures. On 18 May, more activities and businesses opened following social distancing regulations. Phase II in the Lombardy region included the continuation of practicing social distancing until the end of the summer. On 4 June, new containment measures were adopted.

##### Sentiment Analysis

Figure A1 shows the metropolitan city of Milan’s total number of COVID-19 cases, policy measures (local, regional, and national level), and total number of deaths in Lombardy. In Milan, a total of 1,372,402 tweets were collected between 2 March 2020 and 14 June 2020 (blue). The daily fatalities for the Lombardy region are shown from 24 February 2020 (Phase 0) to 14 June 2020 (Phase III) (black).

With regard to fear, the highest frequencies of tweets related to fear were found in the month of May with 2.84% of the total collected tweets when compared to March with 2.42%, April with 2.40%, and June with 2.39%, respectively. Milan Twitter users displayed a total of 2.49% tweet frequency expressing fear. Fear may reflect the beginning of Phase II, which eased many of the measures intended to contain the virus spread.

Second, the highest number of tweets expressing anger was found in the month of May (1.49%) with an average of 1.23% of all tweets, compared to the lowest occurrence of angry tweets in the months of June (1.39%), April (1.38%), and March (1.06%), respectively. Milan Twitter users displayed a total of 1.23% of tweets expressing anger.

While Italy was preparing to reopen after the lockdown ended (3 May), Lombardy and Milan were still suffering an increase in the number of positive cases and deaths. This forced local authorities to introduce special measures, such as a night curfew during weekends, to prevent mass gatherings and irresponsible behavior. Similar to fear, anger began at a high point, but demonstrated somewhat greater volatility in the middle phases, with a slight overall trend toward increased levels over time.

Finally, users on Twitter displayed the highest frequency of tweets related to joy in the month of March with 1.75% of the total collected tweets, compared to June with 1.58%, April with 1.42%, and May with 1.22%. Milan Twitter users displayed a total average of 1.57% tweet frequency related to joy.

##### Pearson’s Correlation Coefficient

To measure the strength and direction of the linear relationship of the study, Pearson’s correlations were employed to test the statistical relationship between Milan’s average number of sentiment tweets related to fear, anger, and joy, COVID-19-positive cases, and total number of deceased at the local and regional levels. Table A1 shows a few significant values. Strong positive correlations existed between the number of total COVID-19 deaths at the regional level and total COVID-19 cases with an r-value of 0.99, and between daily COVID-19 cases and daily COVID-19 deaths with an r-value of 0.83. A strong positive relationship between the total number of angry tweets and tweets related to fear was shown by an r-value of 0.44. There was a moderately positive correlation between the total number of angry tweets and the total number of COVID-19 cases with an r-value of 0.36. In addition, a moderate positive relationship between the total number of angry tweets and total number of COVID-19 deaths is reflected by a slightly higher r-value of 0.37. In contrast, significant strong negative correlations existed between total COVID-19 cases and daily cases with an r-value of −0.58 and daily COVID-19 deaths with daily COVID-19 cases at −0.61.

**Table A1:** Milan Pearson’s correlation coefficient matrix.

Variable	1	2	3	4	5	6	7
1. Anger Tweets							
2. Joy Tweets	−0.24 *						
3. Fear Tweets	0.44 ***	−0.11					
4. Daily COVID-19 Cases	−0.27 **	0.064	−0.14				
5. Total COVID-19 Cases	0.36 ***	−0.19	0.10	−0.58 ***			
6. Daily COVID-19 deaths	−0.17	−0.08	−0.20 *	0.83 ***	−0.41 ***		
7. Total COVID-19 deaths	0.37 ***	−0.19	0.10	−0.61 ***	0.99 ***	−0.43 ***	

Note: characteristics: total COVID-19 cases: total number of COVID-19 cases; daily COVID-19 cases: total number of COVID-19 cases per day; total COVID-19 deaths: total number of deaths; daily COVID-19 deaths: total number of deaths per day; anger: total number of Twitter tweets related to anger; joy: total number of Twitter tweets related to joy; fear: total number of Twitter tweets related to fear. *** means that the *p*-value is less than 0.001; ** means that *p*-value is less than 0.01 but more than or equal to 0.001; and * means that *p*-value is less than 0.05 but more than or equal to 0.01.

#### Appendix A.1.2. Venice, Veneto

##### Region Outlook

Veneto is located in the northeastern part of Italy with a population of about 4.9 million people. It is the fourth most hit region with respect to COVID-19 countrywide. On 18 March, provisions were made for non-scheduled public transport services such as taxis and rental drivers and for atypical services. On 20 March, further measures were made to contain gatherings to prevent the spread of COVID-19. On 24 March, provisions were extended for local public transport of iron, water, and rubber for non-scheduled taxi transport and rentals with a driver and for atypical services. On 3 April, a partial extension of ordinance no. 33 was ordered, and further urgent provisions were adopted to combat the gathering of people in public places open to the public in consideration of the ability of the phenomenon to produce the spread of the infection. On 4 April, further provisions were made to combat the gathering of people as well as the closing of some businesses on 12 and 13 April. On 13 April, urgent measures for the containment and management of epidemiological measures regarding COVID-19 emergencies were made. On 27 April, the first measures were made to ease restrictions, and on 3 May, further provisions regarding travel and preventative measures and the implementation of provisions and Phase II remodeling regarding public transportation. On 4 May, adaptations of previous measures were laid down, and on 17 May, urgent measures were made regarding the containment and management of the epidemiological emergency caused by the COVID-19 virus. On 18 May, rules regarding the remodeling of local public transportation of iron, water, and rubber and for non-scheduled taxis and rentals with drivers and for atypical services were introduced. On 23 May, urgent measures to combat and prevent the spread of COVID-19 were added. On 4 June, new containment measures were adopted.

##### Sentiment Analysis

As shown in Figure A2, a total of 127,251 tweets were collected in Venice between 2 March 2020 and 14 June 2020 (blue). The daily fatalities for the Veneto region are shown from 24 February 2020 (Phase 0) to 14 June 2020 (Phase III) (black).

Users on Twitter produced the highest number of tweets related to fear in the month of June with 2.56% of the total collected tweets when compared to March with 2.35%, April a with t 2.15%, and May with 2.39%, respectively. Venice Twitter users displayed a total average of 2.31% tweet frequency expressing fear. Fear may reflect the beginning of Phase II, which eased many of the measures intended to contain the virus spread. The number of daily new COVID-19 cases and daily fatalities for the Veneto region significantly dropped since the month of May and continued to drop in Phase III. The total number of tweets related to fear increased during the end of Phase II (May), which could be related to the re-opening of public transportation services, businesses, Veneto’s regional border, and new added social distance measures related to anti-COVID-19 policy measures (1 m distance).

Second, the highest number of tweets expressing anger was found in the month of June (1.82%), compared to the lowest occurrence of angry tweets in the months of April (1.29%), May (1.28%), and March (1.08%), respectively. Venice Twitter users displayed a total average of 1.22% tweet frequency expressing anger. The highest number of tweets related to anger was at the end of Phase II during the public transport remodeling, opening of activities and businesses following social distance measures, and the opening of Veneto’s regional borders. The Veneto region is located in close proximity to the Lombardy region where the BLM international protest occurred. As a result, the Veneto region could be highly influenced by the Lombardy region; thus, the total high peak number of angry tweets displayed (red) in Venice could be related to the BLM protest which took place between 5 and 7 June.

While Italy was preparing to reopen after the lockdown ended (3 May), Veneto and Venice were still suffering an increase in the number of positive cases and deaths. This forced local authorities to introduce special measures, such as a night curfew during weekends, to prevent mass gatherings and irresponsible behavior.

Finally, users on Twitter displayed the highest number of tweets related to joy in the month of June at 2.49% of the total collected tweets when compared to March at 1.37%, April at 1.73%, and May at 1.32%. Venice Twitter users displayed a total average of 1.51% tweet frequency related to joy (Figure A2). The highest number of tweets related to joy occurred during the reopening of the Veneto region and lowest number of daily fatalities.

##### Pearson’s Correlation Coefficient

Table A2 shows a few notable correlations. First, significantly strong positive correlations appeared between the number of cumulative positive (COVID-19 cases at the regional level) and deceased (Veneto region) with an r-value of 0.96, between anger and fear at 0.63, and daily positive deaths and cases at 0.56. Moderately positive correlations included anger with total COVID-19 cases at 0.26 and anger with total COVID-19 deaths at 0.30. Very strong negative correlation existed between total deaths and daily COVID-19 positives at −0.67. Moderately strong negative correlations were reflected between total COVID-19 cases and daily COVID-19 positives at −0.49, and between fearful tweets and daily COVID-19 deaths at −0.31.

**Table A2:** Venice Pearson’s correlation coefficient matrix.

Variable	1	2	3	4	5	6	7
1. Anger Tweets							
2. Joy Tweets	−0.05						
3. Fear Tweets	0.63 ***	−0.17					
4. Daily COVID-19 Cases	−0.18	−0.04	−0.27 **				
5. Total COVID-19 Cases	0.26 **	0.14	−0.02	−0.49 ***			
6. Daily COVID-19 deaths	−0.17	−0.07	−0.31 **	0.56 ***	0.08		
7. Total COVID-19 deaths	0.30 **	0.12	0.08	−0.67 ***	0.96 ***	−0.15	

Note: characteristics: total COVID-19 cases: total number of COVID-19 cases; daily COVID-19 cases: total number of COVID-19 cases per day; total COVID-19 deaths: total number of deaths; daily COVID-19 deaths: total number of deaths per day; anger: total number of Twitter tweets related to anger; joy: total number of Twitter tweets related to joy; fear: total number of Twitter tweets related to fear. *** means that the *p*-value is less than 0.001; ** means that *p*-value is less than 0.01 but more than or equal to 0.001.

#### Appendix A.1.3. Turin, Piedmont

##### Region Outlook

Piedmont is located in the northwestern part of Italy and is home to the metropolitan city of Turin, the fourth most populated city in the nation. Contrarily to Lombardy, Piedmont developed a much slower slope in the month of March, but later became the second most hit region countrywide. This can explain the late adoption of containment measures after 21 March. On 23 February, the President of Piedmont issued urgent measures in order to contain the spread of COVID-19. These measures included prohibiting the gathering of large crowds, such as events and schools, and hygienic measures. On 21 March, further restrictive measures for the contaminant of COVID-19 were issued. On 29 March, along with the sale of food and other commercial activities, the sale of stationery and office supplies was also allowed. On 3 April, it was announced that containment measures would be extended until 13 April to further slow the spread of COVID-19. On 6 April, additional measures regarding public transportation were added for the management of COVID-19 emergencies. Additional public health recommendations were added on 7 April to measure body temperature at supermarkets and pharmacies, as well as among employees of the workplace. Containment measures were further extended until 3 May with some exceptions by the Prime Ministerial Decree of 10 April. On 20 April, it was announced that all businesses would be closed on April 25 and 1 May, with the exception of pharmacies, parapharmacies, newsagents, and petrol stations. On 2 May, provisions related to Phase II were given, and on 17 May, provisions for the prevention and management of epidemiological emergencies related to COVID-19 were announced. On 22 May, a new ordinance was introduced requiring the use of masks in areas pertaining to shopping centers as well as the closure of catering and food delivery at 1am. From 2 June, new measures were adopted regarding the use of masks; wearing a mask was mandatory only in residential areas and commercial centers, while it was not mandatory for restaurants and bars guests sitting at tables. On 5 June, professional training and sports activities were authorized. New activities finally opened on 13 June.

##### Sentiment Analysis

As shown in Figure A3, a total of 229,171 tweets were collected in Turin between 2 March 2020 and 14 June 2020 (blue). The daily fatalities for the Piedmont region are shown from 24 February 2020 (Phase 0) to 14 June 2020 (Phase III) (black).

With regard to fear, users on Twitter produced the highest number of tweets related to fear in the month of June at 2.79% of the total collected tweets when compared to May at 2.59%, March at 2.51%, and April at 2.42%, respectively. Turin Twitter users produced a total average of 2.52% tweet frequency expressing fear. Fear may reflect the beginning of Phase II, which eased many of the measures intended to contain the virus spread.

Second, the highest number of tweets expressing anger was found in the month of May (1.46%), compared to the lowest occurrence of angry tweets in the months of June (1.44%), April (1.28%), and March (1.24%), respectively. Turin Twitter users displayed a total average of 1.29% tweet frequency expressing anger.

Finally, users on Twitter produced the highest number of tweets related to joy in the month of April at 1.73% of the total collected tweets when compared to May at 1.37%, March at 1.23%, and June at 1.15%, respectively. Turin Twitter users displayed a total average of 1.37% tweet frequency expressing joy. Joy may reflect the beginning of Phase II, which eased many of the measures intended to contain the virus spread.

##### Pearson’s Correlation Coefficient

Table A3 shows a few notable correlations. There was a very strong positive correlation between the total number of COVID-19 deaths and total number of COVID-19 cases with an r-value of 0.99 as well as between the total number of COVID-19 cases and total number of COVID-19 deaths with an r-value of 0.80. There was also a moderately positive relationship between daily COVID-19 cases and the total number of tweets related to joy with an r-value of 0.38. Additionally, there appeared to be a moderately positive correlation between the total number of tweets related to fear and total number of angry tweets with an r-value of 0.38, which is consistent with Milan’s regional and local data. There was a moderately positive relationship between the total number of COVID-19 deaths and total number of tweets related to joy with an r-value of 0.36. In addition, there was a moderately positive relationship between the total COVID-19 cases and tweets related to anger with an r-value of 0.31. Finally, there was a moderately positive relationship between the total number of COVID-19 deaths and total number of tweets related to anger with an r-value of 0.31. Moderately negatively correlated values included daily COVID-19 cases and total COVID-19 deaths with an r-value of −0.38 and daily COVID-19 cases and total COVID-19 cases at −0.31.

**Table A3:** Turin Pearson’s correlation coefficient matrix.

Variable	1	2	3	4	5	6	7
1. Anger Tweets							
2. Joy Tweets	−0.02						
3. Fear Tweets	0.38 ***	−0.14					
4. Daily COVID-19 Cases	−0.08	0.38 ***	−0.26 **				
5. Total COVID-19 Cases	0.31 **	0.12	0.11	−0.31 **			
6. Daily COVID-19 deaths	−0.02	0.36 ***	−0.20 *	0.80 ***	−0.06		
7. Total COVID-19 deaths	0.31 **	0.08	0.14	−0.38 ***	0.99 ***	−0.14	

Note: characteristics: total COVID-19 cases: total number of COVID-19 cases; daily COVID-19 cases: total number of COVID-19 cases per day; total COVID-19 deaths: total number of deaths; daily COVID-19 deaths: total number of deaths per day; anger: total number of Twitter tweets related to anger; joy: total number of Twitter tweets related to joy; fear: total number of Twitter tweets related to fear. *** means that the *p*-value is less than 0.001; ** means that *p*-value is less than 0.01 but more than or equal to 0.001; and * means that *p*-value is less than 0.05 but more than or equal to 0.01.

#### Appendix A.1.4. Bologna, Emilia-Romagna

##### Region Outlook

Emilia-Romagna is situated in the northwest section of the country, comprising the metropolitan city of Bologna. Relevant measures regarding the containment and management during Phase I included enlargement of restrictive measures to the whole regional territory (9 March), restrictions to administration activities and markets (10 March), measures for supermarkets, take-aways, home and vehicle service activities, private healthcare, and hotel facilities (14 March), the reprogramming of rail transport and public bus services and restrictive provisions for travel (15 March), restrictions on the forms of gathering people and further restrictions in the area and closure of bars and cafés in petrol stations in urban centers (18 March), and extraordinary liquidations to facilitate payments to companies engaged in post-earthquake reconstruction for works performed before the suspension of construction sites for COVID-19 health emergency (15 April). In accordance with the end of the national lockdown, Phase II started on 4 May. The reportable protests included: dealers, professionals and former politicians against restrictions in Bologna (2 May), social and after-school educators on contract, riders, entertainment and logistics porters, tourism workers, tenants, resident doctors in the region with the slogan “Income, health and work: the crisis is paid for by the rich” (8 May), and revolts in a Bologna prison (wounded prisoners and agents, police cars set on fire) (9 May). New containment measures were adopted between 21 May and 23 May to contain the virus spread. On 30 May, production activities were further opened. During Phase III, summer activities for children (15 June) and elderly day centers (17 June) were allowed.

##### Sentiment Analysis

As shown in Figure A4, a total of 73,214 tweets were collected in Bologna between 2 March 2020 and 14 June 2020 (blue). The daily fatalities for the Emilia-Romagna region are shown from 24 February 2020 (Phase 0) to 14 June 2020 (Phase III) (black).

With regard to fear, users on Twitter produced the highest number of tweets related to fear in the month of June at 3.07% of the total collected tweets when compared to May at 2.96%, March at 2.78%, and April at 2.29%, respectively. Bologna Twitter users displayed a total average of 2.69% tweet frequency expressing fear. Fear may reflect the beginning of Phase II, which eased many of the measures intended to contain the virus spread.

Second, the highest number of tweets expressing anger was found in the month of May (1.87%), compared to the lowest occurrence of angry tweets in the months of April (1.40%), June (1.37%), and March (1.36%), respectively. Bologna Twitter users displayed a total average of 1.47% tweet frequency expressing anger.

Finally, users on Twitter produced the highest number of tweets related to joy in the month of May with 2.0% of the total collected tweets when compared to March with 1.91%, April with 1.48%, and June with 1.27%, respectively. Bologna Twitter users displayed a total average of 1.78% tweet frequency expressing joy. Joy may reflect the beginning of Phase II, which eased many of the measures intended to contain the virus spread.

##### Pearson’s Correlation Coefficient

Table A4 shows a very strong positive relationship between the total number of COVID-19 deaths at the Emilia-Romagna regional level and total number of COVID-19 cases with an r-value of 0.99. In addition, there was a very strong positive relationship between the total number of COVID-19 cases and the total number of daily COVID-19 deaths with an r-value of 0.90. There was a strong positive relationship between the total number of tweets related to fear and total number of tweets related to anger with an r-value coefficient of 0.67. This could initially be related to the number of protests that occurred in Italy related to the BLM protest against racism and for social justice. A weak positive relationship could be found in the data between the total number of tweets related to joy and total number of angry tweets with an r-value coefficient of 0.21.

**Table A4:** Bologna Pearson’s correlation coefficient matrix.

Variable	1	2	3	4	5	6	7
1. Anger Tweets							
2. Joy Tweets	0.21 *						
3. Fear Tweets	0.67 ***	0.14					
4. Daily COVID-19 Cases	−0.09	0.06	−0.19				
5. Total COVID-19 Cases	0.12	−0.06	0.07	−0.53 ***			
6. Daily COVID-19 deaths	−0.06	0.04	−0.25 *	0.90 ***	−0.34 ***		
7. Total COVID-19 deaths	0.13	−0.06	0.10	−0.60 ***	0.99 ***	−0.43 ***	

Note: characteristics: total COVID-19 cases: total number of COVID-19 cases; daily COVID-19 cases: total number of COVID-19 cases per day; total COVID-19 deaths: total number of deaths; daily COVID-19 deaths: total number of deaths per day; anger: total number of Twitter tweets related to anger; joy: total number of Twitter tweets related to joy; fear: total number of Twitter tweets related to fear. *** means that the *p*-value is less than 0.001; * means that *p*-value is less than 0.05 but more than or equal to 0.01.

### Appendix A.2. Central Regions

#### Appendix A.2.1. Florence, Tuscany

##### Region Outlook

Tuscany is a region in central Italy with a population of about 3.8 million inhabitants, whose regional capital is Florence. To prevent the spread of coronavirus, Tuscany announced relevant measures with regard to containment and management. Specifically, during Phase I, actions taken involved hygiene and public health measures (8 March), the day centers and guidelines on diagnostic procedures (15 March), domestic municipal waste measures (16 March), approval of plan for the construction of 280 intensive care stations (18 March), indications for primary care, treatments, and recommendations for drug therapy of patients at home affected by COVID-19 (6 April), extraordinary measures to combat and contain the spread of the COVID-19 virus in the area of hygiene and public health in the RSA, RSD, and other social health structures (7 April), additional measures for commercial activities to deal with the spread of the COVID-19 virus across the regional territory (13 April), further measures to manage the epidemiological emergency COVID-19 presented in the field of agriculture, wildlife control, and forestry (14 April), and the maintenance and conservation activities of leather in the industrial district of Santa Croce sull’Arno (24 April), and related activities of companies in the textile sector in the district (26 April). For the following phase, Phase II, the region issued provisions for local public transport on 2 May, and announced the reopening of provisions on 3 May. In addition, containment measures for the spread of the COVID-19 virus in the workplace for the broadcasting sector and further provisions of rapid serological tests were issued on 6 May. Furthermore, a number of libraries and museums in Tuscany were gradually opened after the end of May, and the service time and service were shortened. On 8 June, containment measures were adopted to regulate the virus spread in workplaces, training, and retail facilities. Additional containment measures were imposed between 12 June and 16 June.

##### Sentiment Analysis

As shown in Figure A5, a total of 122,251 tweets were collected in Florence between 2 March 2020 and 14 June 2020 (blue). The daily fatalities for the Tuscany region are shown from 24 February 2020 (Phase 0) to 14 June 2020 (Phase III) (black).

With regard to fear, users on Twitter produced the highest number of tweets related to fear in the month of June with 2.85% of the total collected tweets when compared to May with 2.66%, March with 2.03%, and April with 1.99%, respectively. Florence Twitter users displayed a total average of 2.19% tweet frequency expressing fear. Fear may reflect the beginning of Phase II, which eased many of the measures intended to contain the virus spread.

Second, the highest number of tweets expressing anger was found in the month of June (1.52%), compared to the lowest occurrence of angry tweets in the months of May (1.22%), March (1.13%), and April (1.06%), respectively. Florence Twitter users displayed a total average of 1.15% tweet frequency expressing anger.

Finally, users on Twitter produced the highest number of tweets related to joy in the month of May with 1.49% of the total collected tweets when compared to April with 1.47%, June with 1.40%, and March with 1.29%, respectively. Florence Twitter users displayed a total average of 1.38% tweet frequency expressing joy. Joy may reflect the beginning of Phase II, which eased many of the measures intended to contain the virus spread.

##### Pearson’s Correlation Coefficient

Table A5 shows a very strong positive relationship between total number of daily COVID-19 cases and the total number of daily COVID-19 deaths with an r-value coefficient of 0.77. There was a strong positive relationship between the total number of daily COVID-19 cases and total number of daily COVID-19 deaths with an r-value of 0.62. There was a strong positive relationship between the total number of COVID-19 deaths and total number of tweets related to joy with an r-value of 0.54. In addition, a strong positive relationship was seen between the total number of tweets related to joy and the total number of angry tweets with a coefficient of 0.53. A moderate positive correlation could be seen between the total number of COVID-19 cases and the total daily COVID-19 cases with an r-value of 0.39. Finally, there was a moderate positive relationship between the total number of COVID-19 deaths and the total number of angry tweets related to COVID-19 with a coefficient r-value of 0.32.

**Table A5:** Florence Pearson’s correlation coefficient matrix.

Variable	1	2	3	4	5	6	7
1. Anger Tweets							
2. Joy Tweets	0.53 ***						
3. Fear Tweets	0.07	0.12					
4. Daily COVID-19 Cases	−0.13	−0.49 ***	−0.06				
5. Total COVID-19 Cases	−0.22 *	−0.28 **	0.12	0.39 ***			
6. Daily COVID-19 deaths	−0.12	−0.39 ***	−0.01	0.62 ***	0.77 ***		
7. Total COVID-19 deaths	0.32 ***	0.54 ***	0.13	−0.60 ***	0.11	−0.16	

Note: characteristics: total COVID-19 cases: total number of COVID-19 cases; daily COVID-19 cases: total number of COVID-19 cases per day; total COVID-19 deaths: total number of deaths; daily COVID-19 deaths: total number of deaths per day; anger: total number of Twitter tweets related to anger; joy: total number of Twitter tweets related to joy; fear: total number of Twitter tweets related to fear. *** means that the *p*-value is less than 0.001; ** means that *p*-value is less than 0.01 but more than or equal to 0.001; and * means that *p*-value is less than 0.05 but more than or equal to 0.01.

#### Appendix A.2.2. Rome, Lazio

##### Region Outlook

Lazio is an administrative region of Italy situated in the center, and has approximately 5.9 million inhabitants, which makes it the second most populated region of Italy. In accordance with the national “Stay at Home” campaign, Lazio imposed hygiene and public health measures (8 March) and restrictions for travelers in and out of the region. On 12 March, the regions adopted a tax suspension to relieve the already compromised financial situation. Further containment measures were adopted in the following days, including business hours restrictions (17 March), special measures for voluntary activities to help during the pandemic (20 March), and public transportation measures (24 March). While cases started to decrease in April, Lazio made few concessions, including permits for amateur agriculture and farm animal activities (15 April), opening of beaches, campsites, and shipbuilding areas (17 April), restoration of children’s clothing sales, and in-region transfers and marine activities (24 April). Following the beginning of Phase II, the region reopened all suspended commercial activities on 12 May and adopted measures for the safe reopening of social, cultural, and economic activities following the social distancing measures. Further activities reopened during the second half of Phase II, including production activities (19 May) and social, cultural, and economic activities (27 May). Additional measures to contain the virus spread were adopted during Phase II (29 May–2 June) and Phase III (20 June).

##### Sentiment Analysis

Figure A6 shows the metropolitan city of Rome’s total number of COVID-19 cases, policy measures (local, regional, and national level), and total number of deaths in Lazio. For Rome, a total of 1,899,689 tweets were collected between 2 March 2020 and 14 June 2020 (blue).

With regard to fear, users on Twitter produced the highest number of tweets expressing fear in the month of April with 2.43% of the total collected tweets when compared to March with 2.16%, May with 2.12%, and June with 1.93%, respectively. Rome Twitter users displayed a total average of 2.21% tweet frequency related to fear.

Second, Rome users on Twitter produced the highest number of tweets expressing anger in the month of April at 1.26% frequency with an average of 1.17% when compared to the lowest number of angry tweets in the months of March (1.14%), June (1.11%), and May (1.10%).

Finally, users on Twitter displayed the highest frequency of tweets expressing joy in the month of April with 1.42% of the total collected tweets when compared to April with 1.41%, March with 1.32%, and May with 1.21%. Rome Twitter users displayed a total average of 1.32% tweet frequency expressing joy. As the number of cases decreased, the region eased its measures in April, allowing amateur activities such as farming, outdoor sports and activities, and tourism attractions.

##### Pearson’s Correlation Coefficient

Table A6 shows a very strong positive relationship between the total number of tweets related to joy and total number of angry tweets related to COVID-19 with an r-value of 0.70. There was a strong positive relationship between the total number of COVID-19 cases and the total number of COVID-19 deaths with an r-value of 0.59. A moderate positive relationship was found between the total daily of COVID-19 deaths and the total COVID-19 cases with an r-value of 0.39. In addition, there was a moderate positive relationship between the total number of tweets related to fear and the total number of angry tweets with an r-value of 0.33. Finally, there was a moderate positive relationship between the total number of daily COVID-19 deaths and total number of daily COVID-19 cases with an r-value of 0.31.

**Table A6:** Rome Pearson’s correlation coefficient matrix.

Variable	1	2	3	4	5	6	7
1. Anger Tweets							
2. Joy Tweets	0.70 ***						
3. Fear Tweets	0.34 ***	0.18					
4. Daily COVID-19 Cases	0.03	−0.01	0.10				
5. Total COVID-19 Cases	0.07	0.10	0.07	0.02			
6. Daily COVID-19 deaths	−0.08	−0.14	0.12	0.31 **	0.39 ***		
7. Total COVID-19 deaths	0.10	0.04	0.09	−0.55 ***	0.59 ***	0.12	

Note: characteristics: total COVID-19 cases: total number of COVID-19 cases; daily COVID-19 cases: total number of COVID-19 cases per day; total COVID-19 deaths: total number of deaths; daily COVID-19 deaths: total number of deaths per day; anger: total number of Twitter tweets related to anger; joy: total number of Twitter tweets related to joy; fear: total number of Twitter tweets related to fear. *** means that the *p*-value is less than 0.001; ** means that *p*-value is less than 0.01 but more than or equal to 0.001.

### Appendix A.3. Southern Regions

#### Appendix A.3.1. Naples, Campania

##### Region Outlook

The Campania region is located in the south of Italy. Its capital, Naples, is the third most populated city in the nation. Following massive relocation travels from the north to southern regions before the lockdown, Campania imposed self-quarantine and home isolation for the newcomers (8 March). Stricter measures came along with the “Stay at Home” ordinance, including the shutdown of hairdressers and beauty centers (10 March), fairs and markets (11 March), and parks (12 March). Although the COVID-19 outbreak was mild compared to that in northern regions, Campania was one of the regions with the most stringent containment measures. On 23 March, multiple restrictions were imposed to reorganize car rentals and retailers, food sales, and basic goods activities. On 25 March, the travel ban was further extended to limit unnecessary dwellings. Towards the beginning of Phase II, the region prepared to reopen safely, adopting public transport regulations that followed the social distancing measures (30 April). After the lockdown was lifted, Campania prepared to patrol the region’s borders, fearing that newcomers could have brought a rise in the number of COVID-19-positive cases. After 22 May, the region relaunched the tourism sector, including beach reopening (22 May) and new measures to open hotel and tourism facilities safely (24 May). New policies on premises and alcohol supplies were adopted on 29 May. Further prevention measures were adopted on 5 June and 12 June.

##### Sentiment Analysis

Figure A7 shows the metropolitan city of Naples’s total number of COVID-19 cases, policy measures (local, regional, and national level), and total number of deaths in Campania. For Naples, a total of 263,889 tweets were collected between 2 March 2020 and 14 June 2020 (blue).

With regard to fear, users on Twitter produced the highest number of tweets expressing fear in the month of June with 3.85% of the total collected tweets when compared to May with 2.56%, April with 2.42%, and March with 2.16%, respectively. Naples Twitter users displayed a total average of 2.38% tweet frequency related to fear.

Second, users on Twitter produced the highest number of tweets expressing anger was found in the month of June at 2.02% frequency with an average of 1.24% when compared to the lowest number of angry tweets in the months of April (1.34%), May (1.21%), and March (1.11%).

Finally, users on Twitter displayed the highest frequency of tweets expressing joy in the month of April with 1.55% of the total collected tweets when compared to May with 1.35%, March with 1.33%, and June with 1.25%. Naples Twitter users displayed a total average of 1.39% tweet frequency expressing joy.

##### Pearson’s Correlation Coefficient

Table A7 shows a very strong positive correlation between the total number of tweets related to joy and the total number of angry tweets with an r-value coefficient of 0.88. There was a strong positive relationship between the total daily COVID-19 cases and the total daily COVID-19 deaths with an r-value of 0.65. In addition, there was a strong positive relationship between the total COVID-19 cases and the total number of tweets related to fear with an r-value coefficient of 0.41. A strong positive relationship was found between the total number of COVID-19 cases and total daily COVID-19 deaths with an r-value of 0.41. There was a moderate positive relationship between the total COVID-19 deaths and the total COVID-19 cases with an r-value of 0.38. A moderate positive relationship was found between the total number of daily COVID-19 cases and total number of tweets related to fear with an r-value of 0.31. Additionally, there was a moderate positive relationship between the total number of COVID-19 cases and the total daily COVID-19 cases with an r-value of 0.28. There was a weak negative relationship between the total number of COVID-19 deaths and total number of tweets related to joy with an r-value of 0.26. In addition, there was a weak positive relationship between the total number of COVID-19 deaths and total number of COVID-19 angry tweets with an r-value of 0.24. Finally, there was a weak positive relationship between the total number of tweets related to fear and total number of daily COVID-19 deaths with an r-value of 0.25.

**Table A7:** Naples Pearson’s correlation coefficient matrix.

Variable	1	2	3	4	5	6	7
1. Anger Tweets							
2. Joy Tweets	0.88 ***						
3. Fear Tweets	−0.01	−0.17					
4. Daily COVID-19 Cases	−0.08	−0.16	0.31 **				
5. Total COVID-19 Cases	−0.03	−0.12	0.41 ***	0.28 **			
6. Daily COVID-19 deaths	−0.05	−0.13	0.25 *	0.65 ***	0.41 ***		
7. Total COVID-19 deaths	0.24 *	0.26 **	0.09	−0.46 ***	0.38 ***	−0.21	

Note: characteristics: total COVID-19 cases: total number of COVID-19 cases; daily COVID-19 cases: total number of COVID-19 cases per day; total COVID-19 deaths: total number of deaths; daily COVID-19 deaths: total number of deaths per day; anger: total number of Twitter tweets related to anger; joy: total number of Twitter tweets related to joy; fear: total number of Twitter tweets related to fear. *** means that the *p*-value is less than 0.001; ** means that *p*-value is less than 0.01 but more than or equal to 0.001; and * means that *p*-value is less than 0.05 but more than or equal to 0.01.

#### Appendix A.3.2. Bari, Apulia

##### Region Outlook

Apulia is situated in the south of Italy and is home to the metropolitan city of Bari. Despite the lower number of confirmed cases, the region enforced strict regulations to halt in-region movements during Phase I, especially those associated with tourism purposes and marine activities, and imposed public transport measures (11 April). Containment measures were eased at the end of April, including concessions for amateur agriculture and farm animal activities (17 April) and preparation of outdoor facilities following the social distancing measures (21 April). During Phase II, Apulia prepared to reopen for tourism, including receiving travelers from across the country after 3 June. In accordance with national regulations, the region took measures to safely relaunch the production industry (14 May). Further concessions followed, including the resumption of extracurricular activities (20 May), particularly tailored to the youth segment, and reopening of the region’s amusement parks (24 May). New containment measures were adopted on 2 June in preparation for the reopening of regional borders (3 June). Further tourism activities were opened on 10 June, including spa facilities, wellness centers, tourist guides, cultural, and recreational clubs.

##### Sentiment Analysis

Figure A8 shows the metropolitan city of Bari’s total number of COVID-19 cases, policy measures (local, regional, and national level), and total number of deaths in Apulia. In Bari, a total of 42,221 tweets were collected between 2 March 2020 and 14 June 2020 (blue).

With regard to fear, users on Twitter produced the highest number of tweets expressing fear in the month of June with 3.83% of the total collected tweets when compared to May with 3.24%, April with 2.43%, and March with 2.34%, respectively. Bari Twitter users displayed a total average of 2.61% tweet frequency related to fear.

Second, Bari users on Twitter produced the highest number of tweets expressing anger in the month of June with 2.02% frequency with an average of 1.27% when compared to the lowest number of angry tweets in the months of May (1.63%), April (1.36%), and March (0.95%).

Finally, users on Twitter displayed the highest frequency of tweets expressing joy in the month of April at 1.56% of the total collected tweets when compared to March at 1.249% (1.25%), May at 1.248% (1.25%), and June at 0.98%. Bari Twitter users displayed a total average of 1.33% tweet frequency for tweets expressing joy.

##### Pearson’s Correlation Coefficient

Table A8 shows a strong positive relationship between the total daily COVID-19 deaths and total daily COVID-19 cases at the regional level with an r-value of 0.57. There was a strong positive relationship between the total of COVID-19 deaths and total angry tweets with an r-value of 0.53. Additionally, there was a strong positive relationship between the total number of tweets related to joy and total number of angry tweets related to COVID-19 with an r-value of 0.52. There was a strong positive correlation between the total COVID-19 cases and total daily COVID-19 deaths with an r-value of 0.42. Additionally, there was a strong positive relationship between the total COVID-19 deaths and total COVID-19 number of cases with an r-value of 0.47. A strong positive relationship was found between the total of tweets related to joy and the total number of COVID-19 deaths with an r-value of 0.444. Finally, there was a weak positive relationship between the total COVID-19 cases and total tweets related to fear with an r-value of 0.256.

**Table A8:** Bari Pearson’s correlation coefficient matrix (Regional).

Variable	1	2	3	4	5	6	7
1. Anger Tweets							
2. Joy Tweets	0.52 ***						
3. Fear Tweets	−0.13	−0.30 **					
4. Daily COVID-19 Cases	−0.17	−0.42 ***	0.20 *				
5. Total COVID-19 Cases	0.16	−0.19	0.26 **	0.20 *			
6. Daily COVID-19 deaths	−0.07	−0.28 **	0.17	0.57 ***	0.42 ***		
7. Total COVID-19 deaths	0.53 ***	0.44 ***	−0.03	−0.50 ***	0.47 ***	−0.13	

Note: characteristics: total COVID-19 cases: total number of COVID-19 cases; daily COVID-19 cases: total number of COVID-19 cases per day; total COVID-19 deaths: total number of deaths; daily COVID-19 deaths: total number of deaths per day; anger: total number of Twitter tweets related to anger; joy: total number of Twitter tweets related to joy; fear: total number of Twitter tweets related to fear. *** means that the *p*-value is less than 0.001; ** means that *p*-value is less than 0.01 but more than or equal to 0.001; and * means that *p*-value is less than 0.05 but more than or equal to 0.01.

### Appendix A.4. Islands

#### Appendix A.4.1. Palermo, Sicily

##### Region Outlook

Sicily is the nation’s largest island, located about 3 km off the southwestern coast of Italy. It is also one of the five Special Statute Regions, meaning that it can benefit from special constitutional rights. Since Italy’s nationwide lockdown in response to COVID-19 pandemic, Sicily enforced hygiene and public health measures (8 March) during Phase I. A stringent lockdown was imposed on 11 March, followed by the suspension of all connections to and from the region (13 March). A stricter lockdown strategy was imposed by banning the practice of any outdoor motor activity, closing down all business activities on Sundays, and extending public holidays (3 April) till 13 April. Sicily prepared for easing the measures during Phase II (30 April) followed by revocation of the red areas (1 May). Further regulations with respect to economic, production, public transport, and health preventive measures along with the tourism industry were interchanged as per the Italian Prime Minister’s authorization (17 May). Thus, overall, Phase I and Phase II strategies helped Sicily to limit the spread of the virus while the nationwide lockdown was followed by some internal mobility relaxation during Phase II. At the end of Phase II, while monitoring the situation closely, Sicily’s regional government announced subsidized holidays to attract future tourist visitors to the Mediterranean island to keep the tourism industry ongoing. Additional containment measures were introduced on 2 June in preparation for the reopening of regional borders (3 June).

##### Sentiment Analysis

Figure A9 shows the metropolitan city of Palermo’s total number of COVID-19 cases, policy measures (local, regional, and national level), and total number of deaths in Sicily. For Palermo, a total of 65,035 tweets were collected between 2 March 2020 and 14 June 2020 (blue).

With regard to fear, users on Twitter produced the highest number of tweets expressing fear in the month of March with 2.55% of the total collected tweets when compared to May with 2.48%, June with 2.47% and the lowest number of tweets related to fear in April at 2.23%, respectively. Palermo Twitter users displayed a total average of 2.44% tweet frequency related to fear.

Second, Palermo users on Twitter produced the highest number of tweets expressing anger was found in the month of June with 1.55% frequency with an average of 1.14% when compared to the lowest number of angry tweets in the months of May (1.24%), March (1.11%), and April (1.08%).

Finally, Twitter users displayed the highest number of tweets expressing joy in the month of May with 1.67% of the total collected tweets, compared to April (1.61%), June (1.34%), and the lowest tweets expressing joy in March (1.24%). Palermo Twitter users displayed a total average of 1.42% tweet frequency expressing joy.

##### Pearson’s Correlation Coefficient

Table A9 shows a few notable correlations. There was a strong positive relationship between the total daily COVID-19 deaths and total daily COVID-19 cases with an r-value of 0.70. A strong positive relationship was found between tweets related to joy and angry tweets with an r-value of 0.62. In addition, a strong positive correlation was found between the total number of COVID-19 cases and the total number of COVID-19 death cases with an r-value of 0.60. There was a strong positive relationship between the total number of daily COVID-19 deaths and total number of COVID-19 cases with an r-value of 0.40. There was a weak positive relationship between the total number of COVID-19 cases and the total number of tweets related to fear with an r-value of 0.29. In addition, a weak positive relationship was found between the total number of COVID-19 deaths and total tweets related to fear with an r-value of 0.26. Finally, there was a weak positive relationship between the total number of COVID-19 cases and the total number of tweets related to anger with an r-value of 0.24.

**Table A9:** Palermo Pearson’s correlation coefficient matrix.

Variable	1	2	3	4	5	6	7
1. Anger Tweets							
2. Joy Tweets	0.62 ***						
3. Fear Tweets	−0.12	−0.24 *					
4. Daily COVID-19 Cases	−0.13	−0.06	−0.06				
5. Total COVID-19 Cases	−0.03	−0.19	0.29 **	0.11			
6. Daily COVID-19 deaths	−0.11	−0.09	0.07	0.70 ***	0.40 ***		
7. Total COVID-19 deaths	0.24 *	−0.01	0.26 **	−0.51 ***	0.60 ***	−0.19	

Note: characteristics: total COVID-19 cases: total number of COVID-19 cases; daily COVID-19 cases: total number of COVID-19 cases per day; total COVID-19 deaths: total number of deaths; daily COVID-19 deaths: total number of deaths per day; anger: total number of Twitter tweets related to anger; joy: total number of Twitter tweets related to joy; fear: total number of Twitter tweets related to fear. *** means that the *p*-value is less than 0.001; ** means that *p*-value is less than 0.01 but more than or equal to 0.001; and * means that *p*-value is less than 0.05 but more than or equal to 0.01.

#### Appendix A.4.2. Cagliari, Sardinia

##### Region Outlook

Sardinia is the nation’s second-largest island, located about 188 km off the western coast of Italy. It is also one of the five Special Statute Regions, meaning that it can benefit from special constitutional rights. After the national shutdown of schools and mass gathering, Sardinia imposed hygiene and public health measures (8 March). A strict lockdown was imposed on 11 March, followed by the suspension of all connections to and from the region, as well as air and maritime provisions of non-basic goods (14 March), creating a precedent in the region’s history. Stricter measures to prevent mass gathering were enforced on 24 March, because of the shortage of ICUs and beds in the region’s hospitals. During Phase II, the region relaunched internal tourism through opening of beaches and coastal activities, while it proposed strict regulations for travels from the other Italian regions. After the reopening of regional borders, Sardinia adopted new measures to track travels on/off the island (7 June), including the introduction of travel visas and health reports for tourists. Additional measures on public health and hygiene were introduced on 14 June.

##### Sentiment Analysis

Figure A10 shows the metropolitan city of Cagliari’s total number of COVID-19 cases, policy measures (local, regional, and national level), and total number of deaths in Sardinia. In Cagliari, a total of 32,759 tweets were collected between 2 March 2020 and 14 June 2020 (blue).

With regard to fear, users on Twitter produced the highest number of tweets expressing fear in the month of May with 3.20% of the total collected tweets when compared to June with 2.82%, March with 2.68%, and April with 2.55%, respectively. Cagliari Twitter users displayed a total average of 2.75% tweet frequency related to fear.

Second, Cagliari users on Twitter produced the highest number of tweets expressing anger in the month of March with 1.61% frequency with an average of 1.55% when compared to the lowest number of angry tweets in the months of May (1.52%), April (1.51%), and June (1.32%).

Finally, users on Twitter displayed the highest frequency of tweets expressing joy in the month of June with 2.54% of the total collected tweets when compared to March with 1.60%, May with 1.57%, and April with 1.49%. Cagliari Twitter users displayed a total average of 1.62% tweet frequency for tweets expressing joy.

##### Pearson’s Correlation Coefficient

Table A10 shows some notable correlations. Strong positive relationships existed between the total number of COVID-19 cases and the total number of daily COVID-19 deaths with an r-value of 0.53. There was a moderately positive relationship between the total number of tweets related to joy and total number of angry tweets, with an r-value of 0.39, as well as between the total daily COVID-19 deaths and daily COVID-19 cases, with an r-value of 0.38. There was a weak positive relationship between the total COVID-19 cases and total number of daily COVID-19 cases with an r-value of 0.29. Finally, there was a weak positive relationship between the total number of daily COVID-19 cases and the total number of angry tweets, with an r-value of 0.29. We also found that daily COVID-19 cases and total COVID-19 deaths were strongly and negatively correlated with an r-value of −0.51. There was also a weakly negative correlation between fearful tweets and joyful tweets, with an r-value of −0.28.

**Table A10:** Cagliari Pearson’s correlation coefficient matrix.

Variable	1	2	3	4	5	6	7
1. Anger Tweets							
2. Joy Tweets	0.39 ***						
3. Fear Tweets	−0.24 *	−0.28 **					
4. Daily COVID-19 Cases	0.29 **	−0.13	−0.15				
5. Total COVID-19 Cases	0.09	−0.18	−0.11	0.29 **			
6. Daily COVID-19 deaths	0.12	−0.19	−0.11	0.38 ***	0.53 ***		
7. Total COVID-19 deaths	0.05	0.18	−0.06	−0.51 ***	0.14	−0.14	

Note: characteristics: total COVID-19 cases: total number of COVID-19 cases; daily COVID-19 cases: total number of COVID-19 cases per day; total COVID-19 deaths: total number of deaths; daily COVID-19 deaths: total number of deaths per day; anger: total number of Twitter tweets related to anger; joy: total number of Twitter tweets related to joy; fear: total number of Twitter tweets related to fear. *** means that the *p*-value is less than 0.001; ** means that *p*-value is less than 0.01 but more than or equal to 0.001; and * means that *p*-value is less than 0.05 but more than or equal to 0.01.
